# Disentangling Individual Phases in the Hunted vs. Farmed Meat Supply Chain: Exploring Hunters’ Perceptions in Italy

**DOI:** 10.3390/foods10010174

**Published:** 2021-01-16

**Authors:** Maria Elena Marescotti, Eugenio Demartini, Michael Gibbert, Roberto Viganò, Anna Gaviglio

**Affiliations:** 1 Department of Veterinary Science for Health, Animal Production and Food Safety, University of Milano, Via dell’Università 6, 26900 Lodi, LO, Italy; maria.marescotti@unimi.it (M.E.M.); anna.gaviglio@unimi.it (A.G.); 2 Institute of Marketing and Communication Management, Università della Svizzera italiana, Via G. Buffi 13, CH-6900 Lugano, Switzerland; michael.gibbert@usi.ch; 3 AlpVet, Studio Associato AlpVet, 21052 Busto Arsizio, VA, Italy; r.vigano@alpvet.it

**Keywords:** hunters, hunting activity, wild boar meat, farmed meat, wild game meat, food supply chain management, food perception, survey, risk-assessement

## Abstract

The growing body of literature concerning the hunted wild game meat (HWGM) supply chain is mainly focused on the final consumer, while little is known about upstream production processes. Even though the hunter plays a central role here, it is not well understood how hunters themselves perceive their role in the various phases of the production process. The present study explores Italian hunters’ perception of the HWGM supply chain and compares it to their perception towards the conventional farmed meat supply chain. We distinguish several phases of this production process and find that the final phase related to on-site game dressing is considered problematic, perhaps because hunters perceive themselves as less skilled than professional butchers. The results, in fact, show that hunters prefer hunted products over farmed meat, but that they consider hunted wild boar meat less safe compared to farmed pork. Findings from this study provide a rare glimpse from the inside of the supply chain and reveals the needs for a broad risk assessment analysis on the Italian game meat supply chain. Considering the development of the Italian emerging market of the HWGM, our results also highlight the relevance of training activities on hunters in order to increase the safety and quality of the final product.

## 1. Introduction

To assess the prospective of a food supply chain it is important to investigate both consumers and producers’ opinions and perspectives [1,2]. However, while consumer research is widely used as a tool to explore the market weaknesses and strengths of different food categories, the producers’ side is far less studied [3]. This is especially true in the case of the newly emerging hunted wild game meat (HWGM) supply chain [4,5], where the growing body of literature is mainly focused on consumers [6,7,8,9,10,11,12,13].

Consumer studies have pointed to an extensive list of the determinants of consumption/refusal of HWGM. On the one hand, a growing body of literature revealed the existence of a niche market characterized by a higher willingness to pay for HWGM. These are consumers with positive attitudes towards hunting, with the highest degree of knowledge about HWGM production methods and the greater familiarity towards the product [6,7,8,9,10,11,12,13]. On the other hand, there are segments of consumers with negative attitudes and perception towards HWGM, often directly stemming from a certain skepticism towards the figure of the hunter, who is perceived as a person that does not respect hunting regulations, nor the environment by the average consumer [9].

The sizeable body of prior research on the pivotal figure of the hunter can be summarized by three main foci: (i) understanding the opportunity and constraints for wildlife management; (ii) estimating the risks of wildlife disease spread; (iii) investigating hunters’ opinion about relevant topics related to hunting activity. With reference to the first category of studies (wildlife management practices), hunters’ interviews have been used by several authors in order to understand the hunting pressure, the population density and predicting which species are vulnerable [14,15,16,17,18,19,20,21,22,23]. Other authors used them to develop more successful wildlife conservation plans and hunting regulation, promoting conservation and design public interventions to improve hunting practices [24,25,26,27,28,29,30,31,32,33,34]. Moreover, Kinnell et al. [35] investigated hunters’ willingness to pay to prevent a certain species population decrease and protect ecosystems.

Studies in the second category (wildlife disease spread) interviewed hunters in order to identify change in hunting behavior and hunters’ perception towards wildlife disease such as Chronic Wasting Disease—CWD [36,37,38,39,40,41,42,43,44,45,46,47] or Avian Influenza Virus—AIV [48,49]. Most recently, Tokarska-Rodak et al. [50] developed a survey instruments for the evaluation of hunters’ awareness towards the risks connected to tick bites for preventing infections. 

As for the last category (hunters’ opinions), in the Swedish context, Willebrand [51] investigated the opinion of local hunters towards the development of hunting tourism. Burger et al. [52] and Burger and Sanchez [53] explored hunters’ risk perceptions and environmental concerns to evaluate options and prepare plans for future uses of contaminated lands. Other authors evaluated hunters’ level of satisfaction and perceptions towards the hunting experience in public-access land [54,55,56,57,58] or private land [59]. Focusing on African communities, LeBreton et al. [60] and Friant et al. [61] investigated hunters’ perception of disease risk connected to bush meat contact and HWGM consumption. Finally, several authors take advantage of the survey method to explore hunters’ perception of wildlife [62,63,64,65,66,67,68,69,70].

Despite these advances in the literature, there is still only limited knowledge about upstream production processes and how hunters perceive their own product and evaluate hunting as a food supply chain as well as themselves as food producers. Only few studies take into consideration the hunters as primary producers of the HWGM supply chain. For instance, Gaviglio et al. [71,72] estimated the economic value, the quality and the quantity of the HWGM suitable for the sale by interviewing a sample of Italian hunters and found that the meat of a relevant part of the sample lacks the hygienic and quality standards required for trade. Furthermore, Caro et al. [73] explored the hunters’ perceptions regarding the positive and negative aspects associated with hunting practices and found that Spanish hunters felt misunderstood and even attacked by society. In fact, authors found that hunters perceive themselves as people who love nature, participate in the creation of economic value in local communities and share traditional values of comradeships and friendship. On the other hand, hunters themselves were negatively inclined towards their own community mainly because of someone’s bad manners and hunting practices such as feeling as “machine guns used for shooting animals” or hunting bred and subsequently released (as opposed to ‘wild’) animals. Most importantly for our purposes here, previous studies did not disentangle the individual phases of this production process.

Against this background, the objective of the present paper is to explore hunters’ perception of HWGM supply chain and compare it to their perception towards farmed meat supply chain by separating out the main phases (growth of the animal, culling, and evisceration). To this end, we conducted a survey on the perception of Italian hunters of hunted wild boar (*Sus scrofa*) and farmed domesticated pig (*Sus scrofa domesticus*) supply chains. We focused deliberately on Italy as Italian legislations represents an interesting case study, because hunted wild game hardly enters the Italian market. In fact, while other European countries have effectively implemented the rules Regulations 852, 853 and 854/2004 to place wild game on the market, the Italian operating rules are still unclear and fragmented at a regional level [71]. The lack of clarity on commercial standards implies very high transaction costs along the supply chain, preventing Italian hunters to consider themselves as producers and to consider wild ungulates meat as a food product that can enter the general market [71]. As a consequence, the Italian hunting sector continues to be a very ‘private’ affair with hunted meat consumed by the hunters and their acquaintances [71,72,74]. Nonetheless, the few data collected on Italian production of HWGM in the last decade highlights that hunted meat might represent an attractive alternative to farmed meats. For instance, Ramanzin et al. [75] estimated 6828.7 tons of hunted large wild ungulates meat in the hunting season 2009/2010 in Italy. Focusing on a Northern Italian case study, Gaviglio et al. [71] calculated that hunters donate or discard at least one third of the meat of red deer they harvests in a year, while only 7% is sold to restaurant or local butchers at 6.00 €/kg (gross price) [72]. Finally, the wild boar and domesticated pig have been chosen as case study, because they are easily and directly comparable. In addition to this, wild boar represents the most important wild game species in Italy [75]. 

Considering that producers have the highest level of knowledge of the quality of their own production [3], findings from this study will provide a rare glimpse from the inside of a much-understudied supply chain as well as the basis of a wider and specific risk assessment analysis. Moreover, increasing the knowledge of hunters’ own perception could improve the relationship and cooperation between consumers and producers, in order to further develop the emerging markets of the hunted wild game meat internationally (and in our specific case the Italian market). As such, the remainder of the article is structured as follows. First, we outline material and methods. Next, we examine the results in terms of the individual phases of the supply chain. We conclude the paper with a discussion of our findings and the possible future implications.

## 2. Materials and Methods 

The data analyzed in this study are part of an extensive research on consumers’ attitudes, preferences and knowledge about hunted wild boar (*Sus scrofa*) and farmed domesticated pig (*Sus scrofa domesticus*). Data were collected through an online survey distributed during May and June 2019 via email and web-links to a convenience sample of Italian hunters (the subjects were contacted by: (1) the use of mailing lists of hunters collected during years by the authors of the paper during training courses, workshops and seminars; (2) contacting the heads of several hunting districts asking for the distribution of the survey to their associates.). The final sample consisted of 104 hunters, aged over 18 years old. The survey included a section aimed at collecting the socio-demographic characteristics and HWGM consumption habits of the respondents, and a section aimed at collecting perceptions towards the hunted wild boar meat and the farmed pork meat supply chains (Appendix A). To detect consumption habits, respondents were asked to indicate their frequency of consumption of HWGM from different species (wild boar, red deer, roe deer, chamois) on a 5-point Likert scale ranging from never = 0 to very often = 5. Hunters perceptions towards the different stages of the HWGM and the farmed meat supply chains were detected using 5-point Likert scales (ranging from 1 = “strongly disagree” to 5 = “strongly agree”) ad hoc developed. The final set included 19 items organized into four sub-scales concerning the following stages of the supply chain: animal growth (seven items), culling (four items), evisceration (four items), final product (four items). As product of interest, wild boar was taken into consideration for the HWGM supply chain while pork was used for the farmed meat supply chain. Data were analyzed using IBM SPSS Statistics (SPSS Inc. Chicago, IL, USA). Difference between attitudes towards types of supply chain (hunted wild boar meat and farmed pork meat) were assessed using descriptive analysis and paired *t*-tests. 

## 3. Results

### 3.1. Socio-Demographics and HWGM Consumption of Italian Hunters

The Table 1 provide an overview of the socio-demographic characteristics of the sample of hunters. The majority of the respondents were male, aged between 56 and 65 and not responsible for daily meal purchase. Most of the sample held a high school degree, live in an inland hilly or mountainous area, and have a monthly household income between 2.001 and 4.000€. Concerning the household size, almost two third of the respondents live in households of at least three or a greater number of members. No more than a fifth of the respondents has children under 12 or aged between 13 and 18 in the household.

In Table 2 the respondents’ HWGM consumption habits per species are reported. Consumption frequencies are quite high and differ between species. The most frequently consumed species is wild boar. Furthermore, roe deer meat and red deer meat are consumed often and very often respectively by a third and a fifth of the respondents. Chamois is the species that has been found to be consumed less frequently. In fact, the majority of the sample claimed that they had never consumed it in the last year or that they had consumed it rarely, perhaps because of their natural habitat located in high mountainous alpine areas. This data confirms previous studies indicating that while the consumption of wild game meat in Italy is generally low, in hunters’ families it increases to significant levels [71,75]

### 3.2. Disentangling Perceptions towards Hunted Wild Boar Meat and the Farmed Pork Meat in Different Stages of the Supply Chain 

The following four subparagraphs describe the results of the hunters’ perceptions towards the individual stages of the HWGM and the farmed meat supply chains related to animal growth, culling, evisceration and final product.

#### 3.2.1. Animal Growth—Wild-Life vs. Breeding

**Figure 1:**
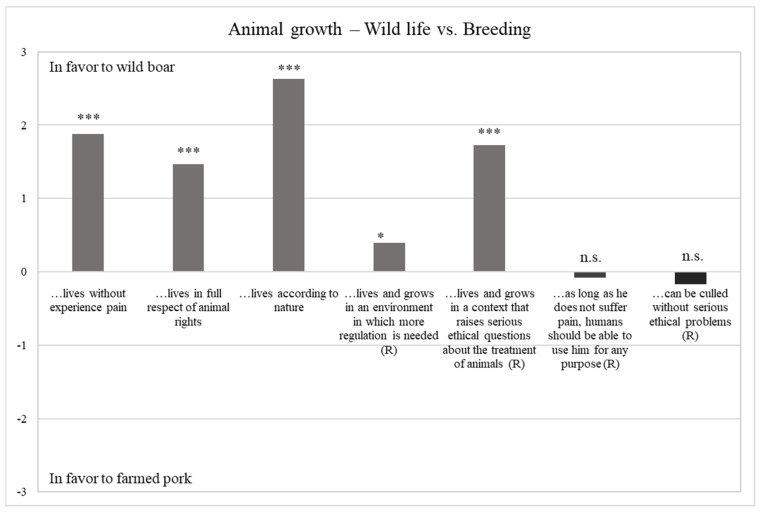
Difference between perceptions towards wild life vs. Breeding. *** Sign. < 0.001; * Sign. < 0.050; n.s. not significant.

Table 3 reports the results of hunters’ perceptions towards the first phase of the supply chain, related to animal growth, while Figure 1 shown the differences between hunters’ perceptions towards wild-life and breeding. Compared to the conventional farmed meat supply chain, the first step of the HWGM supply chain is evaluated more positively by the interviewed sample (mean value = 3.70 vs. 2.57). In fact, hunters believe that wild boar lives freely and according to nature and in a context that does not raise serious ethical questions compared to farmed domesticated pigs. Furthermore, wild boar’s life is perceived as painless respectfull of animal rights. On the contrary, hunters declared that pigs suffer mistreatment during the growth phase and that do not live in full respect of animal rights.

#### 3.2.2. Culling—Hunting vs. Slaughtering

**Figure 2:**
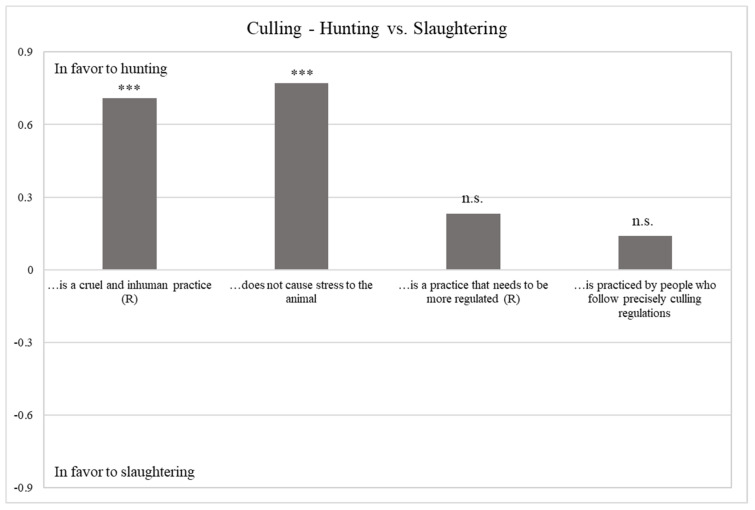
Difference between perceptions towards hunting vs. slaughtering. *** Sign. < 0.001; n.s. not significant.

With reference to the culling phase, as for the animal growth phase, hunters perceive the hunting harvesting method more positively than slaughtering (mean value 3.71 vs. 3.24). Indeed, as summarized in Table 4 the interviewees consider hunting less cruel and inhuman and less stressful for the animal than slaughtering. Overall, the sample agreed that both the hunting and slaughtering do not need stricter regulations and that are practiced by people who follow precisely the legal standards and the good practices indicated by culling regulations (Figure 2).

#### 3.2.3. Evisceration—Hunter vs. Slaughterhouse Operator

**Figure 3:**
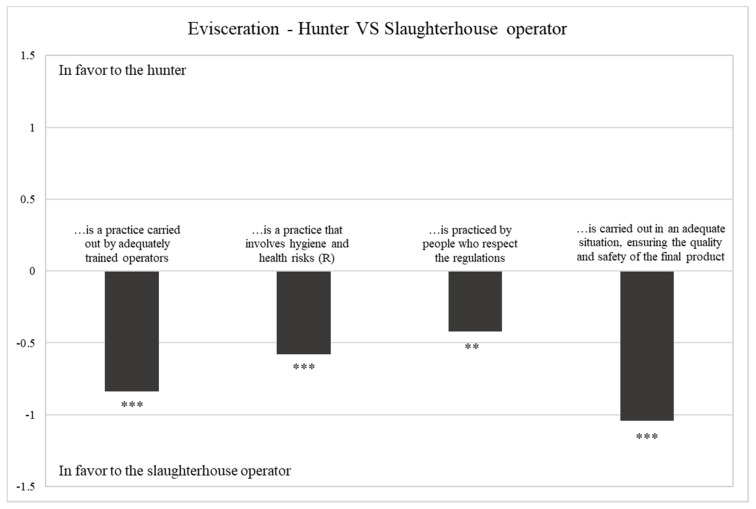
Difference between perceptions towards hunters vs. slaughterhouse operator. *** Sign. < 0.001; ** Sign. < 0.010; n.s. not significant.

The results of hunters’ perceptions towards the evisceration phase of the HWGM and the farmed meat supply chains are reported in Table 5. In contrast to what emerged for the first two steps of the supply chain, respondents showed more positive evaluations for the processing practices carried out by slaughterhouse operators on pork than those carried out by hunters on wild boar (mean value 3.82 vs. 3.10). Slaughterhouse operators are considered more adequately trained, more able to guarantee the quality and safety of the product and more respectful of the regulations. Furthermore, hunters agreed that the processing practices carried out after killing on the hunted wild boar involve higher health and hygiene risks related to incorrect on-site game dressing. In fact, untrained hunters might compromise the quality of game meat mainly due to errors during evisceration, resulting in bacterial contamination, or suboptimal bleeding operations, impeding the correct acidification of meat [4]. As shown in Figure 3 all the investigated statements revealed statistically significant differences between the two supply chains (Figure 3). Considering the composition of the recruited sample, consisting only of individuals who practice hunting, it is worth underlining for emphasis that these results are of particular relevance. Hunters in fact are the primary producers and therefore have the highest level of knowledge about the quality and risks connected to their own production methods [3].

#### 3.2.4. Final product—Wild boar vs. Pork

**Figure 4:**
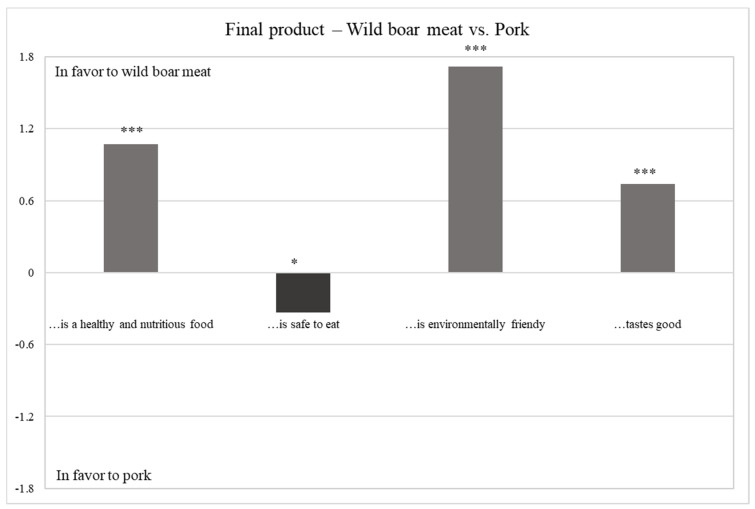
Difference between perceptions towards wild boar meat vs. pork. *** Sign. < 0.001; * Sign. < 0.050 n.s. not significant.

Table 6 and Figure 4 report the results related to hunters’ perceptions towards the final product, namely the hunted wild boar meat vs. the pork. Hunters generally prefer hunted wild boar meat over pork (mean value 4.43 vs. 3.63). In fact, hunted wild boar meat is considered healthier, tastier and more environmentally friendly than domesticated pig meat. On the other hand, it is perceived by hunters themselves as less safe to eat than farmed meat. This finding is consistent with the study performed by Gaviglio et al. [71] indicating that most of the hunted wild game meat lacks the hygienic and quality standards required for trade, mainly due to bad hunting practices [4].

## 4. Conclusions

The current study deliberately focused on how primary producers (rather than final consumers), and specifically hunters, perceive their own product as well as the production process, i.e., how do they evaluate hunting activity and themselves as food producers. In particular, we explored hunters’ perception in the individual phases of the HWGM supply chain and compared it to their perception towards conventional farmed pork supply chain. Italian hunters were considered an interesting case study for two reasons. Firstly, the game meat market seems less developed than what it could be in Italy. In fact, given a certain availability of heads hunted per year and a stable demand, commercial exchanges of local product are extremely limited [71,72,75]. Secondly, the European regulations allows HWGM to be sold, and in fact in other countries such as Austria, Germany, Slovenia and Hungary game meat can be purchased in food retail shops.

As expected, our results pointed to peculiarities that characterize this specific segment compared to the average Italian consumer. In fact, hunters’ preferences are oriented towards the consumption of hunted products, which are preferred over farmed products. Hunted wild boar meat is considered healthier, tastier and more ethical and environmentally friendly than conventional farmed meat. These hunters’ opinion in this phase of the supply chain seem in line with Italian consumers’ perceptions as shown by Demartini et al. [9]. In this research, in fact, half of the sample perceived HWGM as traditional, healthy, tasty and ethical. A similar result is reported in a recent paper by Hartmann and Siegrist [76] that found that German consumers perceive eating HWGM ethically more justifiable than meat produced using conventional methods. Nonetheless, it is worth being underlined that the hunted wild game meat can be positively compared to farmed meats only if hunting practices are conducted by expert shooters and where national/local regulations disincentive hunters’ misconduct. For example, in their seminal review, Hoffman and Wilklund [77] highlighted that culling practices can be less cruel than conventional killing of animal at slaughterhouse, because skilled shooters can harvest game with a single shot, preventing the stress of the prey and even that of other animals. Furthermore, Giacomelli et al. [78] describe a local regulation applied in northern Italy that allows only culling wild boar outside the regular hunting season thereby eradicating hunters’ incentives to artificially release farmed boar just before the hunting season. Finally, Fiala et al. [5] calculated that hunted wild red deer meat footprint is mainly due to the transportation of untrained hunters in the hunting site and concluded that its ecological impact is one-fourth of the beef’s carbon footprint.

On the other hand, somewhat ironically, HWGM is perceived by hunters themselves as less safe to eat. The present research also untangled this effect by separating two phases in this production process. Thus, we found that while hunters’ perceptions towards the first phases of the supply chains (animal growth and culling) where more positive in the case of the HWGM supply chain, their perceptions towards the evisceration phase of the supply chain where more positive towards the conventional farmed meat supply chain. Slaughterhouse operator are considered more adequately trained, more able to guarantee the quality and safety of the product and more respectful of the regulations than hunters. Furthermore, hunters agreed that the processing practices carried out after killing on the hunted wild boar involve higher health and hygiene risks. In fact, according to the literature, the evisceration and bleeding are the most critical on-site dressing operations on games and involve bacterial contamination risks and potential deterioration of the meat quality due to muscle acidification issues [4]. These results show that hunters evaluate themselves in a negative way, in particular owing to the final phases of the production process, reporting overall that they do not trust their own product and consider a product deriving from farmed meat more reliable and safer. Moreover, in line with previous research in the Italian context [71], findings from this study suggest that hunters so far do not consider themselves as producers and reject the idea that wild ungulates meat is a food product that can enter the general market. These results are of key importance for the development of the new emerging HWGM supply chain, highlighting how the training phases of hunters and the communication of quality and safety are essential for the success of the final product.

This study has some limitations. The first limitation is connected to the relatively small sample size, which could affect the representativeness of the results. Nevertheless, hunters’ survey studies and producers’ survey studies in general involve fewer respondents than consumer studies. Moreover, hunters comprise only a small portion of the Italian population, constituted by a very specific segment of people with features that can reasonably be expected to be relatively homogeneous. The second limitation is connected to the self-stated evaluation performed by the hunters. In this regard, the supply chain phase in which they self-assessed their practices worst, is the phase in which there are the greater food safety risk for the final consumer. Therefore, since in some cases hunters evaluated themselves worse than the reality, the self-stated bias does not led to risks, and rather, hunters showed to be very sensitive in the evaluation of the final product in terms of public health.

Given that, as reported in the literature, consumers have positive attitudes towards the consumption of HWGM, but hunters do not trust their own product, future research should perform specific supply chain risk assessment study as well as investigate the mismatch of consumer-producers (i.e., hunters) knowledge and its origin and potential negative consequences in terms of public health. Our finding with regard to the two main phases of the production process (culling vs. eviscerating) could be a useful starting point for these endeavors.

## Figures and Tables

**Table 1:** Socio-demographic characteristics of the sample.

	*n*	%		*n*	%
**Age**			**Household Income (€ per Month)**		
18–25 years	10	10.00	<1000	6	5.77
26–35 years	14	14.00	1000–2000	27	25.96
36–45 years	13	13.00	2001–4000	43	41.35
46–55 years	20	20.00	4001–6000	24	23.08
56–65 years	33	33.00	>6000	4	3.85
66–75 years	9	9.00	**Household Size (Number)**		
over 75 years	1	1.00	1	9	8.65
**Gender**			2	29	27.88
Male	94	90.38	3	32	30.77
Female	10	9.62	4+	34	32.69
**Education**			**Children in the Household 0–12 Years**		
First and secondary school	9	8.65	No	83	79.81
High school	51	49.04	Yes	21	20.19
Bachelor degree	7	6.73	**Children in the Household 13–18 Years**		
Master Degree or higher	37	35.58	No	86	82.69
**Residence Area**			Yes	18	17.31
Coastal	10	9.62	**Responsible for Daily Meal Purchase**		
Inland flat	43	41.35	No	83	79.81
Inland hilly/mountainous	51	49.04	Yes	21	20.19

Number of subjects = 104.

**Table 2:** Wild game meat consumption of the sample.

	Wild Boar		Red Deer		Roe Deer		Chamois	
	*Sus scrofa*		*Cervus elaphus*		*Capreolus* *capreolus*		*Rupicapra* *rupicapra*	
	*n*	%	*n*	%	*n*	%	*n*	%
Never	3	2.88	19	18.27	15	14.42	54	51.92
Rarely	16	15.38	33	31.73	21	20.19	23	22.12
Sometimes	42	40.38	33	31.73	33	31.73	16	15.38
Often	27	25.96	11	10.58	19	18.27	7	6.73
Very often	16	15.38	8	7.69	16	15.38	4	3.85

Number of subjects = 104.

**Table 3:** Animal growth—Wild-life vs. Breeding.

	Wild Boar…		Farmed Pork…		Paired Sample *t*-Test			
					Difference (Wild Boar—Farmed Pork)		*T*	Sign.
	Mean	Std Dev.	Mean	Std Dev.	Mean	Std Dev.		
…lives without experience pain	4.57	0.97	2.68	1.443	1.88	1.77	10.860	0.000
…lives in full respect of animal rights	4.29	1.19	2.82	1.44	1.47	1.82	8.234	0.000
…lives according to nature	4.74	0.74	2.11	1.31	2.63	1.64	16.351	0.000
…lives and grows in an environment in which more regulation is needed (R)	2.82	1.56	2.42	1.34	0.39	2.01	1.998	0.048
…lives and grows in a context that raises serious ethical questions about the treatment of animals (R)	4.03	1.46	2.30	1.37	1.73	1.96	9.019	0.000
…as long as he does not suffer pain, humans should be able to use him for any purpose (R)	2.41	1.56	2.49	1.57	−0.08	1.15	−0.679	0.499
…can be culled without serious ethical problems (R)	3.04	1.66	3.21	1.69	−0.17	1.46	−1.205	0.231

Statements marked with ‘R’ are negative and were reversed for the final scores.

**Table 4:** Culling—Hunting vs. Slaughtering.

	Wild Boar Hunting…		Pork SlaughTering…		Paired Sample *t*-Test			
					Difference (Wild Boar—Farmed Pork)		*T*	Sign.
	Mean	Std Dev.	Mean	Std Dev.	Mean	Std Dev.		
…is a cruel and inhuman practice (R)	4.70	0.71	3.99	1.24	0.71	1.20	6.067	0.000
…does not cause stress to the animal	3.31	1.32	2.54	1.35	0.77	1.72	4.548	0.000
…is a practice that needs to be more regulated (R)	3.02	1.57	2.79	1.33	0.23	1.62	1.452	0.149
…is practiced by people who follow precisely culling regulations	3.81	1.27	3.66	1.25	0.14	1.75	0.841	0.402

Statements marked with ‘R’ are negative and were reversed for the final scores.

**Table 5:** Evisceration—Hunter vs. Slaughterhouse operator.

	Processing Practices Carried Out after Killing on the Hunted Wild Boar…		Processing Practices Carried Out after Killing on the Farmed Pork…		Paired Sample *t*-Test			
					Difference (Wild Boar—Farmed Pork)		*t*	Sign.
	Mean	Std Dev.	Mean	Std Dev.	Mean	Std Dev.		
…is a practice carried out by adequately trained operators	3.38	1.40	4.22	1.02	−0.84	1.50	−5.706	0.000
…is a practice that involves hygiene and health risks (R)	2.30	1.37	2.88	1.49	−0.58	1.36	−4.339	0.000
…is practiced by people who respect the regulations	3.60	1.17	4.02	1.02	−0.42	1.33	−3.251	0.002
…is carried out in an adequate situation, ensuring the quality and safety of the final product	3.13	1.30	4.16	1.04	−1.04	1.55	−6.827	0.000

Statements marked with ‘R’ are negative and were reversed for the final scores.

**Table 6:** Final product—Wild boar meat vs. Pork.

	Hunted Wild Boar Meat…		Farmed Pork Meat…		Paired Sample *t*-Test			
					Difference (Wild Boar—Farmed Pork)		*t*	Sign.
	Mean	Std Dev.	Mean	Std Dev.	Mean	Std Dev.		
…is a healthy and nutritious food	4.60	0.78	3.53	1.16	1.07	1.24	8.773	0.000
…is safe to eat	3.86	1.14	4.18	0.93	−0.33	1.29	−2.588	0.011
…is environmentally friendly	4.57	0.87	2.85	1.21	1.72	1.38	12.760	0.000
…tastes good	4.70	0.71	3.96	1.09	0.74	1.23	6.135	0.000

## Data Availability

Data are available upon request.

## Appendix A. Overview of the Survey Questions

### Appendix A.1. How Often Have You Eat Game Meat of the Following Species in the Last Year?

	**Never**	**Rarely**	**Sometimes**	**Often**	**Very Often**
Wild boar (*Sus scrofa*)	O	O	O	O	O
Red dear (*Cervus elaphus*)	O	O	O	O	O
Roe dear (*Capreolus capreolus*)	O	O	O	O	O
Chamois (*Rupicapra rupicapra*)	O	O	O	O	O

### Appendix A.2. Would You Agree or Disagree with the Following Statements Related to the Hunted Wild Boar Meat versus Farmed Pork Supply Chain?

#### Appendix A.2.1. Supply Chain Phase—Animal Growth

	**Wild Boar…**					**Farmed Pork…**				
	**Strongly Disagree**	**Disagree**	**Neither Disagree** **Nor Agree**	**Agree**	**Strongly Agree**	**Strongly Disagree**	**Disagree**	**Neither Disagree** **Nor Agree**	**Agree**	**Strongly Agree**
…lives with no pain	O	O	O	O	O	O	O	O	O	O
…lives in full respect of animal rights	O	O	O	O	O	O	O	O	O	O
…lives according to nature	O	O	O	O	O	O	O	O	O	O
…lives and grows in an environment in which more regulation is needed (R)	O	O	O	O	O	O	O	O	O	O
…lives and grows in a context that raises serious ethical questions about the treatment of animals (R)	O	O	O	O	O	O	O	O	O	O
…as long as he does not suffer pain, humans should be able to use him for any purpose (R)	O	O	O	O	O	O	O	O	O	O
…can be culled without serious ethical problems (R)	O	O	O	O	O	O	O	O	O	O

#### Appendix A.2.2. Supply Chain Phase—Culling

	**Wild Boar Hunting…**					**Pork Slaughtering…**				
	**Strongly Disagree**	**Disagree**	**Neither Disagree** **Nor Agree**	**Agree**	**Strongly Agree**	**Strongly Disagree**	**Disagree**	**Neither Disagree** **Nor Agree**	**Agree**	**Strongly Agree**
…is a cruel and inhuman practice (R)	O	O	O	O	O	O	O	O	O	O
…does not cause stress to the animal	O	O	O	O	O	O	O	O	O	O
…is a practice that needs to be more regulated (R)	O	O	O	O	O	O	O	O	O	O
…is practiced by people who follow precisely culling regulations	O	O	O	O	O	O	O	O	O	O

#### Appendix A.2.3. Supply Chain Phase—Evisceration

	**Processing Practices Carried Out after Killing on the Hunted Wild Boar…**					**Processing Practices Carried Out after Killing on the Farmed Pork…**				
	**Strongly Disagree**	**Disagree**	**Neither Disagree** **Nor Agree**	**Agree**	**Strongly Agree**	**Strongly Disagree**	**Disagree**	**Neither Disagree** **Nor Agree**	**Agree**	**Strongly Agree**
…is a practice carried out by adequately trained operators	O	O	O	O	O	O	O	O	O	O
…is a practice that involves hygiene and health risks (R)	O	O	O	O	O	O	O	O	O	O
…is practiced by people who respect the regulations	O	O	O	O	O	O	O	O	O	O
…is carried out in an adequate situation, ensuring the quality and safety of the final product	O	O	O	O	O	O	O	O	O	O

#### Appendix A.2.4. Supply Chain Phase—Final Product

	**Hunted Wild Boar Meat…**					**Farmed Pork Meat…**				
	**Strongly Disagree**	**Disagree**	**Neither Disagree** **Nor Agree**	**Agree**	**Strongly Agree**	**Strongly Disagree**	**Disagree**	**Neither Disagree** **Nor Agree**	**Agree**	**Strongly Agree**
…is a healthy and nutritious food	O	O	O	O	O	O	O	O	O	O
…is safe to eat	O	O	O	O	O	O	O	O	O	O
…is environmentally friendly	O	O	O	O	O	O	O	O	O	O
…tastes good	O	O	O	O	O	O	O	O	O	O
